# A case of osmotic demyelination syndrome detected after cesarean delivery with the administration of magnesium sulfate for threatened preterm labor

**DOI:** 10.1186/s40981-020-00376-x

**Published:** 2020-09-08

**Authors:** Yuna Takeshita, Mitsuru Ida, Masahiko Kawaguchi

**Affiliations:** Department of Anesthesiology, Nara Medical University, 840 Shijo-cho, Kashihara, Nara, 634-8522 Japan

To the Editor,

Magnesium sulfate (Mg) is a commonly used tocolytic agent that induces complications such as muscle weakness. The pregnancy-associated osmotic demyelination syndrome (ODS) is rare, although it can be caused by some situations, leading to muscle weakness and quadriplegia [1–3]. In addition, transient neurologic symptoms and cauda equina syndrome following spinal anesthesia for cesarean section could result in gait disorder. With the patient’s consent, we report gait disorder for ODS detected post-cesarean delivery in the patient treated with intravenous Mg as a tocolytic agent.

A 36-year-old primipara was hospitalized at week 26 and day 6 for threatened preterm labor. Then, parenteral ritodrine hydrochloride for tocolysis was administered. However, 21 days later, because of the significant increase of her liver enzymes, it was gradually decreased, and Mg began to be administered at the dose of 1 g/h, following a 4-g loading dose. On day 5 of administration of Mg, she complained ptosis, lower limb muscle weakness, and dizziness. Finally, on day 10, she was unable to walk in the usual way. These symptoms were considered hypermagnesemia-associated adverse events, but the Mg administration rate was increased for tocolysis. However, on day 16 of Mg administration, which is week 32 and day 1, Mg was discontinued due to dyspnea. Then, emergency cesarean delivery was performed under uneventful spinal anesthesia with normal babies. Afterward, despite discontinuing Mg administration, she still was unable to walk in the usual way. On postoperative day 5, the anesthesiologist consulted for gait disorder confirmed that the patient presented with spastic gait and upper-limb ataxia. Further examination showed hyperactivation of her deep tendon reflexes in both the upper and the lower limbs; her brain magnetic resonance imaging (MRI) confirmed the diagnosis of ODS (Fig. 1). Afterward, she started rehabilitation and was discharged walking independently, on postoperative day 17. In these clinical courses, with serum sodium concentration ranged from 135 mg/dL to 142 mg /dL, although she had no baseline serum magnesium concentrations, the serum Mg concentration reached the maximum concentration of 5.1 mg/dL 12 days after administration and it decreased by 1.7 mg/dL, 2 days after discontinuing Mg administration.

**Fig. 1:**
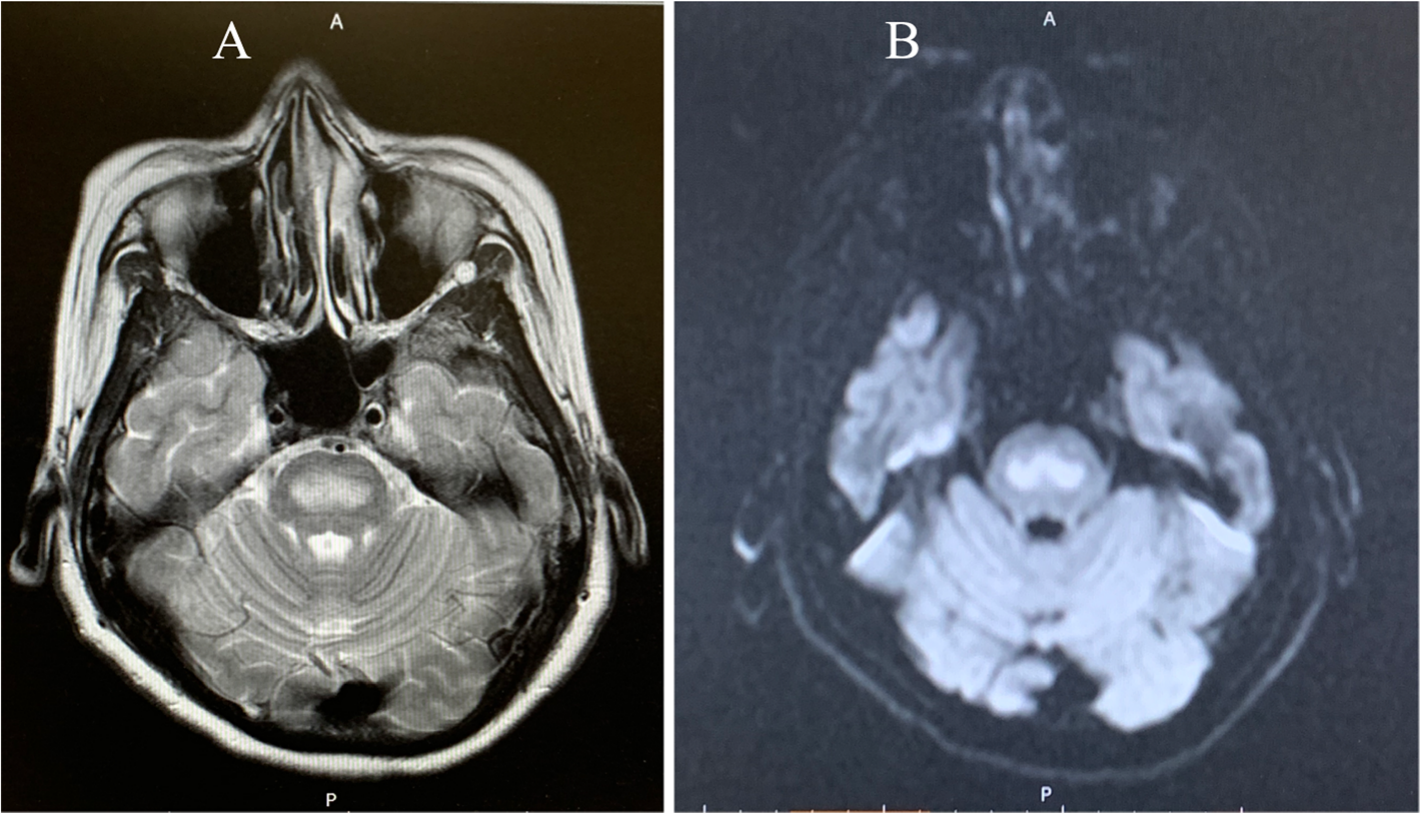
Cerebral magnetic resonance imaging showing hypersignals in the central pons and medial cerebellum in both the T2 weighted (**a**) and the diffusion-weighted images (**b**)

A brain MRI is the gold standard to diagnose ODS, but it takes at least 2 weeks after disease onset for findings to become positive [4]. This explains that ODS had already occurred before cesarean delivery. Considering the onset, the contribution of Mg to the pathogenesis of ODS could be suspected [2]. The exact mechanism has been unclear, but glial cells may be susceptible to the toxic effects of hypermagnesemia [5]. We selected spinal anesthesia, but careful selection of anesthetic techniques may be required because the impact of spinal anesthesia on ODS is poorly documented. Suspecting ODS is the first step toward diagnosing this syndrome and ODS must be considered when patients receiving Mg present with gait disorder and lower limb muscle weakness regardless of serum concentration and administration rate of Mg. Additionally, frequent examinations of deep tendon reflex may be useful in making a differential diagnosis between hypermagnesemia with hyperreflexia and ODS with hyporeflexia, respectively, because positive signs in MRI are not timely presented.

## Data Availability

Not applicable
